# Embodied perspective-taking indicated by selective disruption from aberrant self motion

**DOI:** 10.1007/s00426-016-0755-4

**Published:** 2016-02-22

**Authors:** Mark R. Gardner, Chloé Stent, Christine Mohr, John F. Golding

**Affiliations:** 1 Department of Psychology, University of Westminster, 115 New Cavendish Street, London, W1W 6UW UK; 2 Institute of Psychology, University of Lausanne, Bâtiment Geopolis, Quartier Mouline, 1015 Lausanne, Switzerland

## Abstract

Spatial perspective-taking that involves imagined changes in one’s spatial orientation is facilitated by vestibular stimulation inducing a congruent sensation of self-motion. We examined further the role of vestibular resources in perspective-taking by evaluating whether aberrant and conflicting vestibular stimulation impaired perspective-taking performance. Participants (*N* = 39) undertook either an “own body transformation” (OBT) task, requiring speeded spatial judgments made from the perspective of a schematic figure, or a control task requiring reconfiguration of spatial mappings from one’s own visuo-spatial perspective. These tasks were performed both without and with vestibular stimulation by whole-body Coriolis motion, according to a repeated measures design, balanced for order. Vestibular stimulation was found to impair performance during the first minute post stimulus relative to the stationary condition. This disruption was task-specific, affecting only the OBT task and not the control task, and dissipated by the second minute post-stimulus. Our experiment thus demonstrates selective temporary impairment of perspective-taking from aberrant vestibular stimulation, implying that uncompromised vestibular resources are necessary for efficient perspective-taking. This finding provides evidence for an embodied mechanism for perspective-taking whereby vestibular input contributes to multisensory processing underlying bodily and social cognition. Ultimately, this knowledge may contribute to the design of interventions that help patients suffering sudden vertigo adapt to the cognitive difficulties caused by aberrant vestibular stimulation.

## Introduction

Spatial perspective-taking, the ability to adopt spatial relationships from another person’s point of view, is important for many everyday social interactions, such as demonstrating how to do a task or giving directions. Evidence is emerging that the mental transformations involved in aligning one’s own body orientation to that of a third party (Parsons, 1987; Zacks, Rypma, Gabrieli, Tversky, & Glover, 1999; Blanke et al., 2005) are embodied; they appear to involve the mental simulation of not only somatosensory (Gardner & Potts, 2010; Kessler & Rutherford, 2010; Kessler & Thomson, 2010), but also vestibular information (Deroualle, Borel, Devèze, & Lopez, 2015; Falconer & Mast, 2012; van Elk & Blanke, 2014). To date, evidence for vestibular involvement has focussed on facilitation effects during mild, spatially congruent, vestibular stimulation (Deroualle et al., 2015; Falconer & Mast, 2012; van Elk & Blanke, 2014; see Palla & Lenggenhager, 2014). However, if the vestibular system contributes to efficient spatial perspective-taking, disruption of normal vestibular processing would be expected to lead to impaired perspective-taking performance. The aim of the present experiment was therefore to examine the vestibular contribution to spatial perspective-taking, by assessing whether aberrant vestibular stimulation resulting from whole body motion leads to selective disruption to perspective-taking as measured by an own body transformation task (OBT) in which participants have to imagine being in the visuo-spatial position of a depicted figure (e.g., Arzy, Thut, Mohr, Michel, & Blanke, 2006; Blanke et al., 2005; Parsons, 1987; Zacks et al., 1999).

Spatial perspective-taking appears to share brain resources with those normally deployed in processing somatosensory and vestibular information. For instance, imagined transformations of one’s spatial perspective so that it aligns with that of an avatar was facilitated if the body posture of the participant was congruent with the required direction of imagined rotation (Kessler & Thomson, 2010). Similarly, perspective-taking in the OBT task was facilitated when judgements about the hand with which a schematic figure is holding an object correspond with the participant’s dominant hand (Gardner & Potts, 2010). The hypothesis that vestibular processing also contributes to perspective-taking receives support from functional similarities between perceived, and imagined, self-rotation. For example, subdural electrical stimulation of the right angular gyrus has been found to lead to a range of illusory own-body perceptions, including the illusion of self motion, perceived changes in body orientation (out of body experiences) and disruption to the body schema (Blanke, Ortigue, Landis, & Seeck, 2002). Furthermore, imagined self-rotation about the body’s longitudinal axis (yaw plane) elicited directionally consistent nystagmus, an ocular response normally contingent upon vestibular stimulation (Rodionov, Zislin, & Elidan, 2004). These various embodiment effects suggest that imagined self-rotation and the perception of actual self-rotation are performed by overlapping brain systems.

Three recent experiments provide more direct evidence pertaining to this hypothesis, by showing that mild vestibular stimulation may selectively facilitate imagined self-rotation (Deroualle et al., 2015; Falconer & Mast, 2012; van Elk & Blanke, 2014). Falconer and Mast (2012) manipulated afferents from the semicircular canals through caloric vestibular stimulation (CVS) to provide a sensation of rightward body rotation. This stimulation selectively enhanced performance in a task requiring mental transformations of one’s own body in roll (about an anterior-posterior axis); stimulation did not affect tasks requiring mental transformations of objects or body parts. In their experiment, van Elk and Blanke (2014) stimulated the vestibular system through passive self-rotation in a motorised chair. This stimulation selectively facilitated perspective-taking in an OBT task in the form of a congruency effect between the direction of actual and imagined self-motion. A similar congruency effect has been reported for a visual perspective-taking task by Deroualle et al. (2015). Thus, in each of these experiments, facilitation occurred when vestibular stimulation induces a congruent sensation of self-motion to that imagined when performing the task (Palla & Lenggenhager, 2014). These selective facilitation effects provide convincing evidence that vestibular processing relates to mental transformations of the body, by excluding alternative explanations relating to motivation or domain general resources.

If the vestibular system contributes to spatial perspective-taking, disruption of normal vestibular function would be expected to lead to selective impairment to performance. Consistent with this proposition, an impaired ability to perform mental rotation of one’s own body in roll has been found for patients with vestibular loss (Grabherr, Cuffel, Guyot, & Mast, 2011), and for degraded vestibular input occurring when healthy individuals were assessed under microgravity (Grabherr et al., 2007; see Grabherr & Mast, 2010). Administration of galvanic vestibular stimulation (GVS) has been found to disrupt mental transformation in the roll plane for participants having adopted an own body transformation strategy, and not for those having employed object-based transformations (Lenggenhager, Lopez, & Blanke, 2008). Furthermore, participants subjected to a diverse battery of mental tasks were found to perform more poorly for a navigational task (as well as a test of short-term spatial memory) during supra-threshold GVS (Dilda, MacDougall, Curthoys, & Moore, 2012). Taken together, these findings provide converging evidence that uncompromised vestibular resources are necessary for efficient mental transformation of one’s own body in the roll plane. However, it remains unclear whether such disruption extends to spatial perspective-taking, and imagined self-rotation about the yaw axis. Such transformations are habitually performed, pervasive in social interaction, and consequently potentially less likely to require deliberate mental transformations (Samson, Apperly, Braithwaite, Andrews, & Bodley Scott, 2010; Tversky & Hard, 2009).

An alternative method to examine the role of vestibular processing in spatial perspective-taking in healthy individuals is to investigate task costs resulting from complex vestibular sensations induced by motion that do not occur in the natural environment. Such “aberrant” vestibular stimulation, resulting from passive whole body motion and/or active movement of the head, has been found to disrupt cognitive performance (Furman, Redfern, Fuhrman, & Jennings, 2012; Gresty, Waters, Bray, Bunday, & Golding, 2003; Gresty, Golding, Lu, & Nightingale, 2008; Johnson, 1956). This disruption is typically brief, lasting less than one minute (Gresty & Golding, 2009), and may be alleviated by continued exposure to the stimulus or by overtraining with the cognitive task (Gresty et al., 2008). The breadth of cognitive tasks disrupted, including those drawing upon attention (e.g., Johnson, 1956) or memory (e.g., Webb, Estrada, & Kelley, 2012), indicate that to some extent general attentional resources may be disrupted by vestibular stimulation (see Gresty & Golding, 2009). Whereas experiments that compare tasks with high spatial and low spatial load provide evidence for differential disruption to spatial processing (Furman et al., 2012; Gresty et al., 2003, 2008; Gresty & Golding, 2009). Whether impairment is global, or restricted to spatial processing, is thought to be attributable to methodological differences between studies such as the strength of the vestibular stimulus, and the type of spatial processing drawn upon by the cognitive task (Furman et al., 2012). However, research to date has not attempted to isolate specific components of spatial cognition, such as the own body mental transformations involved in perspective-taking, using tasks matched for difficulty.

The present experiment was therefore designed to examine whether aberrant vestibular stimulation caused by actual body motion results in selective disruption to perspective-taking performance. Participants were randomly allocated to perform either the OBT task (Blanke et al., 2005), as a test of perspective-taking that involves mental own body transformations about the yaw axis, or a control task of equivalent difficulty, requiring reconfiguration of spatial mappings from one’s own visuo-spatial perspective (the “Transpose” task, Gardner & Potts, 2011). These mental tasks were performed both under stationary conditions, and immediately after aberrant vestibular stimulation resulting from Coriolis motion (Parmet & Gillingham, 2002). Performance was examined in one minute bins to capture disruption that may dissipate within the first minute post-stimulus (Gresty & Golding, 2009). If vestibular processing contributes to spatial perspective-taking, vestibular stimulation would be predicted to result in poorer performance compared with the stationary condition for the OBT task, but not for the control task. Whereas, if disruption were due to domain general factors, such as the allocation of attentional resources, disruption would be expected to be non-selective.

## Methods

### Participants

In total, 39 student volunteers from the University of Westminster took part in this study (31 females; 22.9 ± 6.5 years). They reported being healthy, with intact vestibular function and not under any current medication, and had normal or corrected to normal vision. All scored beneath the 75th percentile on a motion sickness susceptibility questionnaire (Golding, 2006) indicating that there were no highly susceptible individuals. Participants gave informed consent before testing commenced. The experimental procedure was approved by the local (University of Westminster) ethics committee, and was therefore performed in accordance with the 1964 Declaration of Helsinki.

### Vestibular stimulus

Aberrant vestibular stimulation was provoked by “Coriolis motion” produced by the participant actively tilting their head during passive whole body rotation in yaw (Benson, 1999). This manoeuvre results in the vertical canals receiving an abrupt starting stimulus whenever they are brought into the plane of rotation and the horizontal canals receiving an abrupt stopping stimulus whenever they are taken out of the plane of rotation. At the same time, the otoliths signal both tilt with respect to gravity, and a Coriolis force resulting from their small radial displacement from the axis of yaw rotation (Gresty et al., 2008). This complex vestibular stimulus does not occur in the natural environment and has the capacity to disrupt cognitive performance (Gresty et al., 2008).

Coriolis motion was brought about in the following manner. Participants sat upright, safely restrained in a chair that was motorized to rotate about an Earth vertical axis. A fabric cabin surrounding the chair excluded extraneous visual input. Once the chair had been gradually brought to a constant rotational velocity of 60 °/s (clockwise), participants performed a series of discreet head tilts by moving their head from upright towards four stops located orthogonally around the headrest of the chair. They were prompted to do so for 30 s by pre-recorded audio instructions; e.g., “Forward … Return … Left … Return … Back …Return… Right… Return etc.”. As our objective was not to induce motion sickness, participants were asked to report any motion sickness symptoms on a 6-point scale immediately after vestibular stimulation (1 = no symptoms; 2 = initial symptoms, e.g., stomach awareness but no nausea; 3 = mild nausea; 4 = moderate nausea; 5 = severe nausea; 6 = vomiting; Gresty et al., 2008).

### Perspective-taking and spatial control tasks

Both spatial tasks were implemented using E-Prime experiment generator software (Schneider, Eschman, & Zuccolotto, 2002), running on a laptop which was securely mounted to the chair. The “OBT” task (Own Body Transformation task; Blanke et al., 2005) was employed as a test of spatial perspective-taking, while the “Transpose” task was employed as a spatial control task of equivalent difficulty performed from one’s own visuo-spatial perspective (Gardner & Potts, 2011). The general methodology used for each task was adapted from that reported previously (Gardner & Potts, 2011, Experiments 1A and 3), and summarised below.

**Fig. 1:**
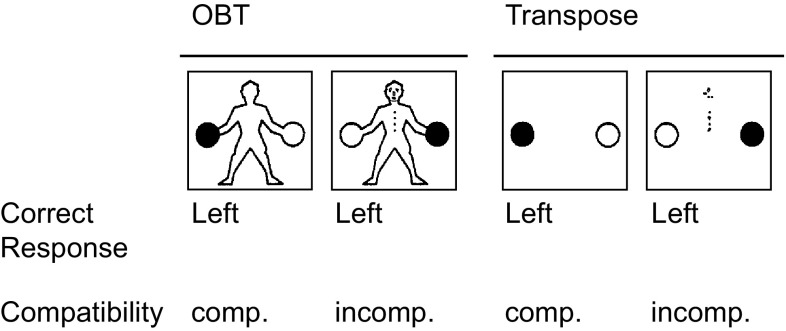
Illustration of the relations between stimulus, response, and stimulus–response compatibility as a function of task for left hand trials (right hand not depicted)

For the OBT task, the stimuli each depicted a schematic human figure holding a black ball in one hand and a white ball in the other (see Fig. 1). The black ball was either in the figure’s left or right hand and the figure could be facing either toward or away from the participant, and always in an upright orientation (in contrast to the otherwise similar “Manikin” test used in Human Factors research: Benson & Gedye, 1963). Front- and back-view stimuli shared the same outline, and were distinguished only by elements indicating a front-view (facial features and buttons). Participants were instructed to imagine themselves in the body position of the figure, in order to judge whether the black ball was being held by the figure’s left or right hand by pressing the corresponding response keys. Throughout testing, participants rested the index finger of each hand on the ipsilateral response key (e.g., their left index finger on the left response key). Consequently, the correct response was compatible with the location of the ball on the screen for back-view stimuli, and incompatible with the location of the ball on the screen for front-view stimuli.

For the Transpose task, stimuli were black and white balls, presented at the same size and angular separation as for the OBT task, but without an accompanying figure (see Fig. 1). For “cue-present” stimuli, which occurred on half the trials, the balls were accompanied by an abstract visual cue comprised of the same eight elements that distinguish front- and back-view stimuli in the OBT task (facial features and buttons) arranged in scrambled configuration. On “cue-absent” trials, participants were required to respond to the location of the black ball as it appeared from their own perspective by pressing the corresponding key. Whereas, on cue-present trials, participants were instructed to transpose left and right when responding. Consequently, the correct response was compatible with the location of the ball on the screen for cue-absent stimuli, and incompatible with the location of the ball on the screen for cue-present stimuli. Thus, the Transpose task involved spatial mappings of equivalent difficulty to the OBT task, but without the perspective-taking requirement.

For both tasks, each trial commenced with a black fixation cross presented centrally for 1400 ms against a white background. This was immediately followed by the stimulus which was displayed until a response had been made up to a maximum of 2100 ms. The stimulus was followed by visual feedback on whether the response was correct or incorrect, which was presented for 1500 ms before being replaced by the fixation cross for the following trial.

### Experimental procedure

Participants were randomly allocated to perform either the “OBT” (own body transformation; Blanke et al., 2005), or the spatial control task (“Transpose” task; Gardner & Potts, 2011). The between-subjects design was chosen to eliminate anticipated carry-over effects had participants performed both tasks (Gardner, Brazier, Edmonds, & Gronholm, 2013). Whilst the chair was stationary, participants first practiced their designated cognitive task. There were 44 practice trials presented in a single block. Participants were instructed to respond as accurately and as fast as possible. They also rehearsed the audio-cued head movements employed to provoke Coriolis motion.

The experiment proper comprised four blocks of mental task trials. These were time-limited, terminating after 2 min had elapsed, and time stamped so that they could be binned into the first and second minutes post-stimulus. Vestibular Stimulus was a within participant manipulation. All participants performed two consecutive blocks whilst the chair was stationary (denoted ‘stationary’), and two consecutive blocks immediately after aberrant vestibular stimulation provoked by Coriolis motion (denoted ‘motion’). The order in which these two conditions were administered was counterbalanced between subjects, and a 5 min break was introduced between conditions to allow for any effects of vestibular stimulation to dissipate.

For the motion condition, the chair was maintained at a constant velocity for approximately 6 min, within which time two cycles of the following procedure were administered—head movement (30 s) followed by a block of mental task trials (120 s) with head stationary. A 30 s interval was interspersed between the two cycles. For the stationary condition, two blocks of the same mental task were administered interspersed with the same intervals, but with the chair stationary and the participant’s head maintained in the resting position. After every block of mental task trials, participants were asked to rate perceived effort relative to the practice block on a 7-point scale (a lot, moderately, mildly less/more effort, or no difference).

## Results

Incomplete data were obtained from two female participants; one due to a data acquisition error, and one because the participant withdrew part way through the protocol. One male participant who did not comply with instructions for the mental tasks and performed at chance (error rate = 50 %) was also excluded from the analysis. For the remaining participants (*N* = 36, 29 female), rates of errors were low (*M* = 4.4, SD = 4.1 %). Motion (Coriolis stimulation) tended to provoke only mild symptoms on the 6-point motion sickness symptom scale (*M* = 2.2, SD = 0.79, range 1–3.5). No difference was found between the sensations reported by participants carrying out the OBT task (*M* = 2.1, SD = 0.90), and those carrying out the Transpose control task (*M* = 2.3, SD = 0.68), *t* (34) = 0.63.

### Perspective-taking and spatial control task performance

Since we were interested in the general efficiency of mental task processing, we employed the standard composite measure “inverse efficiency” (IE, see Townsend & Ashby, 1978), rather than separate measure of response time (RT, ms) and error rate (ER, %). IE is RT divided by the proportion correct (i.e., (100 − ER)/100), and can be more informative than separate analyses of RT and ER when, as was the case here, these variables change in unison and ER <10 % (Bruyer & Brysbaert, 2011). Trials from both blocks were amalgamated, binned into the first and second minute, and means were computed for each condition. Only RTs for correct responses were used. These data are summarised in Table 1, with RT and ER also reported for the sake of transparency.

**Table 1:** Mean response time (ms), error rate (%), inverse efficiency (IE, ms) and perceived effort (scale score, relative to practice) as a function of vestibular stimulation (stationary vs. motion) for both the OBT and transpose tasks

	OBT		Transpose	
	Stationary	Motion	Stationary	Motion
Response times (ms)				
1st min, incompatible	778 (112)	825 (135)	753 (126)	764 (124)
1st min, compatible	710 (122)	764 (126)	641 (107)	644 (90)
2nd min, incompatible	763 (118)	776 (128)	763 (137)	745 (124)
2nd min, compatible	699 (167)	701 (115)	640 (104)	643 (107)
Error rate (%)				
1st min, incompatible	1.9 (3.4)	6.9 (6.8)	5.4 (8.1)	4.8 (7.8)
1st min, compatible	2.9 (4.6)	8.6 (11.2)	3.8 (7.1)	3.8 (6.4)
2nd min, incompatible	2.4 (6.0)	4.7 (7.2)	4.0 (6.1)	7.6 (10.7)
2nd min, compatible	2.2 (2.9)	2.4 (4.1)	4.6 (5.0)	3.6 (5.6)
IE (inverse efficiency, ms)				
1st min, incompatible	795 (122)	895 (182)	801 (149)	804 (124)
1st min, compatible	733 (124)	860 (246)	671 (127)	673 (106)
2nd min, incompatible	785 (132)	822 (168)	801 (172)	824 (219)
2nd min, compatible	714 (179)	722 (141)	675 (135)	669 (120)
Effort (scale, −3 to +3)	−0.1 (1.1)	0.0 (0.9)	−0.2 (1.3)	0.1 (1.3)

Response times, error rates and IE are further segregated as a function of duration since vestibular stimulation (first, and second minute post stimulus) and spatial compatibility. Standard deviations are in parentheses

The data in Table 1 appear to indicate disruption to performance caused by Coriolis motion that was limited to the first minute of testing, and was restricted to the OBT task. This disruption appeared to be similarly present for RT and ER, as well as IE. The IE data were subjected to a 4-way mixed model ANOVA in which task (OBT vs. transpose) was a between-subject factor, and time (1st minute vs. 2nd minute), vestibular stimulus (stationary vs. motion), and compatibility (compatible vs. incompatible) were within-subject factors. As expected, this revealed a main effect of compatibility, *F*(1, 34) = 29.8, *p* < .001, $\eta_{p}^{2}$ = 0.467, indicating poorer performance for trials requiring a spatial transformation (i.e., incompatible, *M* = 816 ms) than those not requiring a transformation (i.e., compatible, *M* = 715 ms). The magnitude of this difference was similar for the stimulus–response remapping demanded by the Transpose control task as for the perspective transformations demanded by the OBT task (compatibility × task interaction, *F*(1, 34) = 3.55, *p* = .068, $\eta_{p}^{2}$ = 0.095). Furthermore, the main effect of Task was not statistically significant, *F*(1, 34) = 1.64, *p* = .208, $\eta_{p}^{2}$ = 0.046, consistent with the two mental tasks being of equivalent difficulty.

**Fig. 2:**
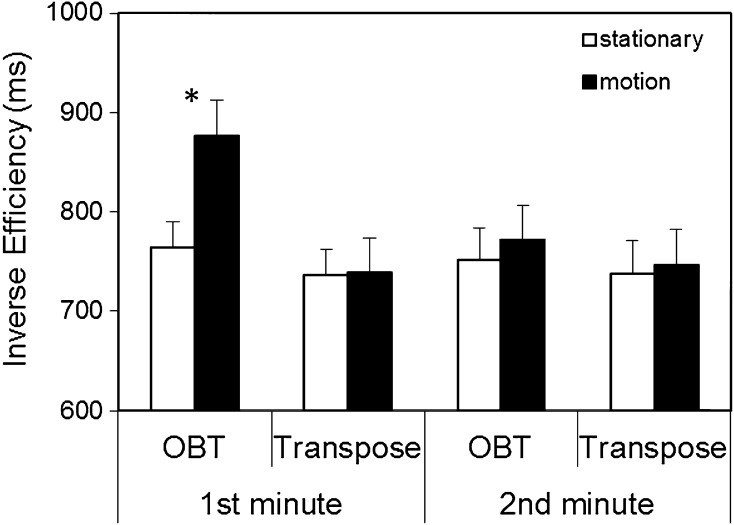
Mean inverse efficiency (ms) for both the OBT and transpose tasks as a function of vestibular stimulation (stationary vs. motion) during the first and second minute post-stimulus. *Error bars* indicate standard error of the mean. *Asterisk* indicates statistically significant simple effect, *p* < .005

A statistically significant main effect of Vestibular Stimulus, *F*(1, 34) = 5.55, *p* = .024, $\eta_{p}^{2}$ = 0.140, indicated disruption to performance caused by Coriolis motion, and a main effect of time, *F*(1, 34) = 6.22, *p* = .018, $\eta_{p}^{2}$ = 0.155, was in keeping with an impairment in performance that was time-limited. An interaction between time and task, *F*(1, 34) = 8.66, *p* = .006, $\eta_{p}^{2}$ = 0.203, implies that this impairment may have been task specific. Although the 3-way interaction between vestibular stimulus, time and task was not significant (at alpha 0.05), *F*(1, 34) = 3.55, *p* = .068, $\eta_{p}^{2}$ = 0.095, further examination of the question of whether impairment was task specific and/or contingent upon motion is warranted. These interactions are illustrated in Fig. 2, with compatibility collapsed given that it did not interact with any other variable.

Figure 2 appears to show temporary disruption in performance measured by IE that was contingent upon Coriolis motion, and restricted to the OBT task. Task specific disruption by motion was examined by a pair of 2-way within-subject task (OBT vs. transpose) × vestibular stimulus (stationary vs. motion) ANOVAs, one at each minute. For the first minute, there was a significant main effect of task, *F*(1, 34) = 4.65, *p* = .038, $\eta_{p}^{2}$ = 0.120. There was also a main effect of vestibular stimulus, *F*(1, 34) = 7.66, *p* = .009, $\eta_{p}^{2}$ = 0.184, that was moderated by task, *F*(1, 34) = 7.09, *p* = .012, $\eta_{p}^{2}$ = 0.173. Simple effect analyses revealed that disruption resulting from motion during the first minute was present for the OBT task, *t*(17) = 3.30, *p* = .004, but not for the transpose task, *t*(17) = 1.06, *p* = .303.

The selective disruption of performance was restricted to the first minute: analysis for the second minute revealed neither a main effect of vestibular stimulus, *F* < 1, $\eta_{p}^{2}$ = 0.006, nor an interaction between vestibular stimulus and task, *F* < 1, $\eta_{p}^{2}$ = 0.003. The main effect of task also was not significant, *F* < 1, $\eta_{p}^{2}$ = 0.016.

This temporary impairment to OBT task performance appears not to be affected by the figure’s orientation, as coded by the compatibility variable. Inspection of the IE data presented in Table 1 suggests that responses to both incompatible/front-view stimuli (difference, *M* = 100, SD = 133), and compatible/back-view stimuli (*M* = 127, SD = 199) contributed to this difference. Post hoc tests revealed a statistically significant difference both for front-view stimuli, *t*(17) = 3.19, *p* = .01, and back-view stimuli, *t*(17) = 2.71, *p* = .03.

### Effort

Ratings of effort for the mental tasks were examined in order to assess whether the effects of motion on RT or PE might be attributed to reduced motivation under vestibular stimulation rather than to reduced cognitive efficiency. Participants rated how much extra effort they invested for experimental trials in relation to practice trials (seven point scale −3 to +3). These data, presented in Table 1, appear to indicate no discernible difference between tasks, neither for the motion nor the stationary conditions. These impressions were confirmed by 2-way mixed ANOVA in which task (OBT vs. transpose) was a between-subject factor, and vestibular stimulus (stationary vs. motion) was a within-subject factor, which revealed no main effect of task, *F* < 1, vestibular stimulus, *F* < 1, nor interaction between these factors, *F* < 1.

## Discussion

The present experiment examined vestibular involvement in perspective-taking by evaluating whether aberrant vestibular stimulation brought about by head movements during passive whole body rotation selectively impaired perspective-taking performance. We found that vestibular stimulation disrupted perspective-taking assessed by the OBT task, but not spatial responding from one’s own perspective assessed by the Transpose control task. Disruption was most pronounced in the first minute post stimulus, in line with the short-term effects of vestibular stimulation on cognitive performance previously reported (Gresty & Golding, 2009; Gresty et al., 2008). The selective disruption to perspective-taking performance was not attributable to a range of potential confounds; the cognitive demands of the OBT and transpose tasks were comparable, as indicated by inverse efficiency scores. Furthermore, these groups were not found to differ in their subjective experience of Coriolis motion, nor in reported effort, suggesting that these findings were not an artefact of differences in motivation or severity of the vestibular stimulus. Thus, the results of this experiment demonstrate disruption to cognitive performance that was selective to perspective-taking as measured by the OBT task.

These selective disruption effects are consistent with vestibular involvement in spatial perspective-taking. The control task was a spatial choice-reaction time task of equivalent difficulty to the OBT task, involving the same mixture of spatially compatible and incompatible S-R mappings, but without the perspective-taking requirement. Non-selective effects would have been expected had vestibular stimulation merely distracted participants, or disrupted attentional resources or interfered with spatial cognition more generally. This evidence is consistent with recent findings that GVS impairs imagined changes in self-orientation in a navigation task (Dilda et al., 2012), and that passive self-motion facilitated directionally congruent imagined self-motion (van Elk & Blanke, 2014) and visual perspective-taking (Deroualle et al., 2015). The present experiment complements these findings, demonstrating additionally that uncompromised vestibular resources are required for efficient perspective taking.

At least two proposals for how the vestibular system might contribute to spatial and social cognition may help to account for our findings. One proposal is that imagined spatial transformations of one’s own perspective are instantiated through the mental simulation of the mechanisms involved in perceiving actual self-motion, including vestibular processing (Deroualle & Lopez, 2014; Palla & Lenggenhager, 2014). This proposal has received strong support from experiments demonstrating that vestibular stimulation facilitates spatially congruent mental transformations of the whole body about the roll (Falconer & Mast, 2012), or yaw axis (van Elk & Blanke, 2014; Deroualle et al., 2015). A second proposal is that vestibular stimulation contributes to multisensory spatial coding relating to the bodily self (Aspell, Lenggenhager, & Blanke, 2009), which could underpin self-other processing in a range of social domains, such as relating one’s own perspective to that of another (Deroualle & Lopez, 2014; Lopez, 2013; Pfeiffer, 2015). Vestibular input is thought to be particularly important compared to other senses by helping to distinguish between movement occurring to “I” (the subject of experience), to another person, or to the environment (Deroualle & Lopez, 2014). This proposal receives support from a range of abnormal bodily cognitions that have been found to result from disruptive vestibular stimulation, including distortions to somatosensory perception (Ferrè, Bottini, & Haggard, 2011) and body awareness (Ferrè, Vagnoni, & Haggard, 2013; Lopez, Schreyer, Preuss, & Mast, 2012b), as well as the higher order experience of depersonalisation (Jáuregui-Renaud, Sang, Gresty, Green, & Bronstein, 2008; Yen Pik Sang, Jauregui-Renaud, Green, Bronstein, & Gresty, 2006; see Lopez, 2013, for a review). Thus, in the present experiment, the complex vestibular stimulation provided by Coriolis motion may have disrupted spatial perspective-taking by depleting the vestibular resources available to mentally simulate self-motion and/or to integrate multisensory bodily codes.

These two accounts are potentially distinguishable by the degree to which disruption is affected by the viewpoint of the figures employed for the OBT task (front- vs. back- view stimuli). If simulated self-rotation was disrupted by vestibular stimulation, performance on the OBT task should have been impaired predominantly for the front-view stimuli that involve a 180° mental self-rotation. Whereas, if comparison of self and other perspective was disrupted by diminished multisensory integration of bodily codes, impairment of performance should not be viewpoint dependent, with performance similarly affected for back- and front-view stimuli. Our data provide support for the latter prediction. Inverse Efficiency scores for back-view stimuli in the first minute following motion were substantially greater than under the stationary condition, and interactions involving the front- vs back-view (compatibility) factor were not significant. The present experiment therefore provides evidence to support the view that vestibular resources contribute to perspective-taking at least partly by facilitating multisensory spatial coding relating to the bodily self (Aspell et al., 2009; Deroualle & Lopez, 2014; Lopez, 2013; Pfeiffer, 2015). This interpretation would predict that aberrant vestibular stimulation would similarly disrupt self-other processing in a range of social domains.

The design employed in the present study does not rule out the possibility that uncompromised vestibular resources are important for spatial transformations more generally; i.e., the possibility that Coriolis motion disrupts performance on object-based mental rotation as well as perspective-taking tasks (variations of paradigms as for instance reported in Zacks et al., 1999; Blanke et al., 2005). There is some limited evidence from prior research for such a generalised effect. For instance, while CVS has been found to influence object-based mental transformations (Mast, Merfeld, & Kosslyn, 2006), more recent work contrasting an imagined self-rotation task with an object based control has found facilitatory effects of CVS that were selective to imagined self-rotation (Falconer & Mast, 2012). Similarly, vestibular disease has been found to disrupt object based transformations (Péruch et al., 2011), although not to the same extent as imagined self-rotation (Grabherr et al., 2011). In general, it seems likely that the degree to which disruption is selective to a cognitive domain, rather than global, will be determined by the extent that vestibular resources have been depleted (Furman et al., 2012). Our favoured interpretation of the present experiment is that effects of aberrant vestibular stimulation probably were selective to perspective-taking. This is based upon the finding that Coriolis motion disrupted performance in the OBT task, not only for the front-view stimuli (which involved perspective-taking and a spatial discrepancy), but also the back-view stimuli (which involved perspective-taking, but no spatial discrepancy). In future work, this interpretation could be tested by employing an object-based mental rotation task of similar difficulty to the OBT task, as an alternative control.

A methodological point about the perspective-taking processes measured by the OBT task may be drawn from the foregoing evidence for a vestibular contribution to performance in this task. It has previously been suggested that domain general response selection processes and spatial compatibility effects alone could account for performance in this task (Gardner & Potts, 2011), and other tasks that measure perspective-taking via laterality judgments (May & Wendt, 2013; see also Braithwaite & Dent, 2011). However, in contrast to such views, the disruption caused by vestibular stimulation in the present experiment did not extend to the Transpose task, which controlled for the domain general processes required to inhibit pre-potent spatially compatible responses. Similarly, the selective facilitation to perspective-taking arising from passive self-motion was reported to be independent of spatial compatibility (van Elk & Blanke, 2014). These selective effects of vestibular stimulation imply that OBT task performance cannot be reduced to spatial compatibility.

The present results lend support for a separable embodied perspective-taking process that is distinct from perspective-taking achieved through the reconfiguration of spatial relationships from one’s own perspective (Gardner et al., 2013; Gronholm, Flynn, Edmonds, & Gardner, 2012; May & Wendt, 2012). In this respect, our findings are consistent with previous work also indicating an embodied mechanism that recruits sensorimotor resources (Becchio, Del Giudice, Dal Monte, Latini-Corazzini, & Pia, 2013; Conson et al., 2014; Furlanetto, Gallace, Ansuini, & Becchio, 2014; Gianelli, Farnè, Salemme, Jeannerod, & Roy, 2011; Kessler & Rutherford, 2010; Kessler & Thomson, 2010). One proposal is that embodied and non-embodied processes are in fact distinct routes modulated by strategy (Gardner et al., 2013; see also Crescentini, Fabbro, & Urgesi, 2014; Kaiser et al., 2008). Indeed, neuroimaging evidence indicates that performing the OBT task more dominantly recruits the area around the temporo-parietal junction than the processing of spatial relationships from one’s own perspective, as if in a mirror, which tends to recruit the extrastriate body area (Arzy et al., 2006). A recent coordinate-based activation likelihood estimation meta-analysis places the major vestibular regions to the restroinsular cortex, the parietal operculum and posterior insula (Lopez, Blanke, & Mast, 2012a), thus, areas within the temporal and parietal lobe rather than the occipital lobe. Although strategy was not measured in the present experiment, it should be noted that strategy has previously been found to moderate the effects of vestibular stimulation (Lenggenhager, Lopez, & Blanke, 2008; van Elk & Blanke, 2014), so fruitful avenues for further research include assessing whether strategy similarly moderates either the neural correlates of spatial perspective-taking, or the effects of aberrant vestibular stimulation.

In conclusion, this experiment demonstrates selective impairment of perspective-taking by aberrant vestibular stimulation. This finding has three main implications. Firstly, it contributes converging evidence for a role of vestibular processing in mental transformations of the body (Falconer & Mast, 2012; van Elk & Blanke, 2014) by demonstrating that uncompromised vestibular resources are necessary for efficient perspective-taking. Secondly, it lends support to the hypothesis that vestibular input facilitates self-other comparison by contributing to the multisensory representation of self (Deroualle & Lopez, 2014; Pfeiffer, 2015), as well underpinning mental simulation of sensory changes involved in self motion (Falconer & Mast, 2012; Palla & Lenggenhager, 2014; van Elk & Blanke, 2014; Deroualle et al., 2015). Thirdly, on a methodological note, this evidence for a specialised embodied mechanism undermines the view that performance in tasks requiring laterality judgments can be accounted for solely in terms of domain general processes (Gardner & Potts, 2011; May & Wendt, 2013). We propose that tasks assessing mental transformations of one’s own body through space may prove useful for future research that seeks to elucidate how best to adapt to aberrant vestibular stimulation that result either from vestibular disease (e.g., vertigo), or challenging environments (e.g., aerospace).
